# Amino Acid Transporter Genes Are Essential for *FLO11*-Dependent and *FLO11*-Independent Biofilm Formation and Invasive Growth in *Saccharomyces cerevisiae*

**DOI:** 10.1371/journal.pone.0041272

**Published:** 2012-07-26

**Authors:** Rasmus Torbensen, Henrik Devitt Møller, David Gresham, Sefa Alizadeh, Doreen Ochmann, Eckhard Boles, Birgitte Regenberg

**Affiliations:** 1 Department of Biology, University of Copenhagen, Copenhagen, Denmark; 2 Center for Genomics and Systems Biology, New York University, New York, New York, United States of America; 3 Department of Biology, New York, New York University, New York, United States of America; 4 Institute of Molecular Biosciences, Goethe University Frankfurt, Frankfurt am Main, Germany; Texas A&M University, United States of America

## Abstract

Amino acids can induce yeast cell adhesion but how amino acids are sensed and signal the modulation of the *FLO* adhesion genes is not clear. We discovered that the budding yeast *Saccharomyces cerevisiae* CEN.PK evolved invasive growth ability under prolonged nitrogen limitation. Such invasive mutants were used to identify amino acid transporters as regulators of *FLO11* and invasive growth. One invasive mutant had elevated levels of *FLO11* mRNA and a Q320STOP mutation in the *SFL1* gene that encodes a protein kinase A pathway regulated repressor of *FLO11*. Glutamine-transporter genes *DIP5* and *GNP1* were essential for *FLO11* expression, invasive growth and biofilm formation in this mutant. Invasive growth relied on known regulators of *FLO11* and the Ssy1-Ptr3-Ssy5 complex that controls *DIP5* and *GNP1*, suggesting that Dip5 and Gnp1 operates downstream of the Ssy1-Ptr3-Ssy5 complex for regulation of *FLO11* expression in a protein kinase A dependent manner. The role of Dip5 and Gnp1 appears to be conserved in the *S. cerevisiae* strain ∑1278b since the *dip5 gnp1* ∑1278b mutant showed no invasive phenotype.

Secondly, the amino acid transporter gene *GAP1* was found to influence invasive growth through *FLO11* as well as other *FLO* genes. Cells carrying a dominant loss-of-function *PTR3^647::CWNKNPLSSIN^* allele had increased transcription of the adhesion genes *FLO1, 5*, *9*, *10*, *11* and the amino acid transporter gene *GAP1*. Deletion of *GAP1* caused loss of *FLO11* expression and invasive growth. However, deletions of *FLO11* and genes encoding components of the mitogen-activated protein kinase pathway or the protein kinase A pathway were not sufficient to abolish invasive growth, suggesting involvement of other *FLO* genes and alternative pathways. Increased intracellular amino acid pools in the *PTR3^647::CWNKNPLSSIN^*-containing strain opens the possibility that Gap1 regulates the *FLO* genes through alteration of the amino acid pool sizes.

## Introduction

Nitrogen is a vital metabolite in living organisms and nitrogen metabolism governs major developmental decisions in *Saccharomyces cerevisiae*. For example, nitrogen starvation triggers diploid cells to undergo meiosis [1] and can lead to quiescence of both haploid and diploid cells [2]. Low concentrations of nitrogen induce pseudohyphal growth through elongated growth and polar budding [3] while propagation of haploid cells on rich complex medium leads to biofilm formation and invasive growth [4], [5].

Biofilm, pseudohyphal growth and haploid invasive growth are dependent on the cell wall adhesin gene *FLO11* (*MUC1*), which confers cell-surface adhesion [6], [7]. Cellular access to nitrogen is linked to *FLO11* expression through the cyclic AMP-protein kinase A (cAMP-PKA) pathway and the general transcription factor Gcn4 [8], [9]. However, a central question is how the yeast cell senses extracellular nitrogen in the form of amino acids and the signal is transmitted to activation of *FLO11.*

Nitrogen in the form of ammonium induces transcription of *FLO11* via the high-affinity ammonium transporter Mep2 and several observations support the hypothesis that Mep2 is an ammonium receptor [8], [10]. However, the ability to transport ammonium appears to be essential for the signaling function of Mep2 [11] suggesting that Mep2 is a transceptor or transports ammonium to an intracellular receptor. Mep2 is proposed to induce expression of *FLO11* via the cAMP-PKA pathway, based on the findings that exogenous cAMP, dominant active *RAS2* or *GPA2* alleles are able to restore pseudohyphal differentiation in a Δ*mep2/*Δ*mep2* strain [8].

Lorenz and Heitman observed that pseudohyphal growth on proline and glutamine-based medium was not dependent on *MEP2* [8] and hypothesized that amino acid transporters could have a function similar to Mep2. In the current work we have explicitly tested the role of amino acid transporters in the regulation of *FLO11*, since these transporters have not yet been shown to play a role in biofilm formation, pseudohyphal or invasive growth.

Glutamine transport is mediated by Gnp1, Agp1, Gap1 and Dip5 [12]–[15] while proline is transported by Put4 and the general amino acid transporter Gap1 [16]. Transcription of *GNP1*, *AGP1* and *DIP5* is induced by extracellular amino acids through the Ssy1-Ptr3-Ssy5 (SPS) complex and deletion of *SSY1* leads to a 5–15 fold reduction in *DIP5* and *GNP1* expression [13], [17], [18]. Counter intuitively, deletion of *SSY1* or *PTR3* lead to increased invasive growth in the Σ1278b strain background [17] while the opposite would be expected if invasive growth only depended on *GNP1*, *AGP1* or *DIP5*. A cause of the invasive growth in the *ssy1* and *ptr3* mutants may be increased expression of *GAP1* and higher levels of intracellular amino acids.

Here we investigated the importance of the amino acid transporters Gap1, Dip5 and Gnp1 in regulation of *FLO11-*dependent invasive growth. We hypothesize that (i) the general amino acid permease Gap1 is essential for invasive growth and *FLO11* expression under conditions where *GAP1* is highly expressed and (ii) that Gnp1 and Dip5 are essential for *FLO11* dependent invasive growth in conditions where *GAP1* is not expressed.

## Results

### Nitrogen-limited *S. cerevisiae* Populations Evolve Invasive Growth

**Figure 1:**
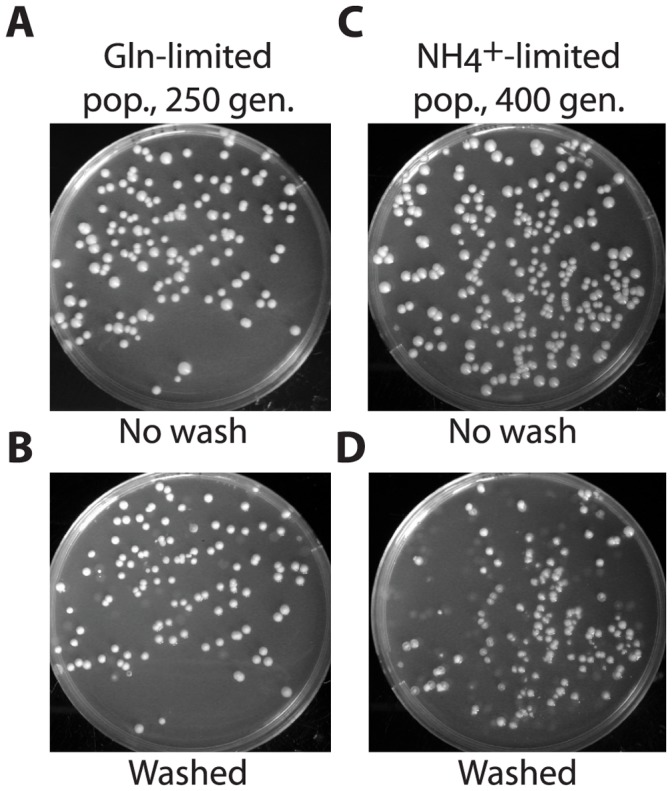
Invasive growth of haploid descendants after prolonged nitrogen limitation. Descendants of CEN.PK113-7D in A, glutamine-limited culture, and C, ammonium-limited culture, grown on rich complex medium (YPD) for 3 days at 30°C and flushed with water to determine the proportion of adhering clones (B and D). Of descendants from the glutamine-limited population, 80% were invasive on YPD, while in the two ammonium-limited populations, 40% and 26% percent had become invasive (n>200).

To investigate the role of amino acids transporters for invasive growth and *FLO11* regulation, we initially tested the phenotype of *gnp1*, *dip5* and *gap1* mutants in the Σ1278b strain background but neither of these single gene deletions led to the loss of the invasive growth phenotype (Fig. S1). In an alternative approach we selected invasive mutants and used these as background for further analysis of amino acid regulation of *FLO11*. Invasive mutants appeared in populations of the normally noninvasive *S. cerevisiae* (CEN.PK113-7D) when propagated under nitrogen limitation (Fig. 1A–D). Clones were isolated from two haploid CEN.PK113-7D populations of 10^10^ cells after 250 generations in continuous bioreactors, limited for ammonium or glutamine respectively.

### Clones Sfl1^Q320STOP^ and Ptr3^647::C…N^ Induce Invasive Growth

**Figure 2:**
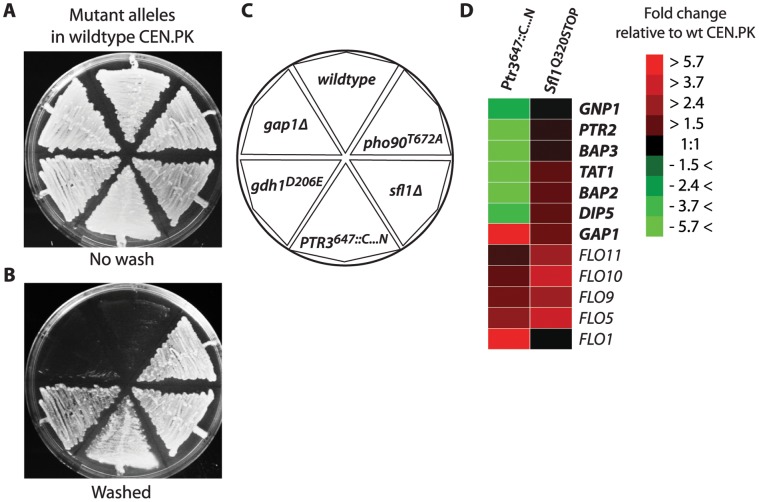
Mutant alleles restore invasive growth in wildtype strain and invasiveness correlates with increased *FLO11* mRNA. Mutations from invasive descendants of glutamine- and ammonium-limited populations were reconstituted in the wildtype strain (A) and tested for invasive growth (B). Distribution of strains with *pho^T672A^* (RB389), *sfl1Δ* (RB595), PTR3^647::C…N^ (RB567), *gdh^D206E^* (RB484), *gap1Δ* (RB318) (C). mRNA levels for FLO adhesion genes (italic) and amino acid permease genes (italic bold) are shown for glutamine-limited mutants Ptr3^647::C…N^ and Sfl1^Q320STOP^ after growth to mid-exponential phase in liquid YPD (D). Presented are log2-transformed ratios of transcripts. The color code bar illustrates fold-change in mRNA in mutants relative to the wildtype strain CEN.PK113-7D. For the entire clustering of genes with more than 2-fold altered mRNA levels see Fig. S2.

To characterize clones from nitrogen-limited populations, we compared the genomes of six clones to the wildtype using DNA tiling arrays, revealing 12 to 88 single nucleotide polymorphisms (SNPs) per genome. We characterized the SNPs that affected ORFs by PCR and Sanger sequencing and verified up to two mutations in ORFs per clone (Table 1). Mutant alleles identified in the descendants were subsequently introduced into the wildtype strain to test if these caused the invasive growth phenotype. All gave rise to invasive phenotypes, except for a deletion of the general amino acid permease gene *GAP1* (Fig. 2A–C).

**Table 1:** Peptide modifications in six clonal isolates from prolonged nitrogen-limited populations.

Gene	Glutamine-limited clones			Ammonium-limited clones		
	I	II	III	IV	V	VI
	Invasive	Invasive	Non-invasive	Non-invasive	Invasive	Invasive
*GAP1*			Δ*gap1*	S388P	Δ*gap1*	
*MEP2*				G352S		
*PHO90*	T672A					
*SFL1*	Q320 STOP					
*PTR3*		647::CWNKNPLSSIN				
*GDH1*					D206E	
*SAM4*			Synonymous			

Denoted are the protein modifications resulting from mutations in the clonal isolates. The corresponding mutations are described in Material and Methods.

We focused our work on two of the invasive clones from the glutamine-limited population. The clone Sfl1^Q320STOP^ (Table 1) carries two mutant alleles: the nonsense mutation *sfl1^C958T^* that leads to a truncation of Sfl1 at residue 320 and the *pho90^A2014G^* mutation that substitutes threonine with alanine at residue 672 of Pho90. Clone Ptr3^647::C…N^ (Table 1) carries a partial substitution of the *PTR3* ORF with the long terminal repeat (LTR) of a Ty1 element from residue 1941 of *PTR3.* This leads to substitution of the 31 C-terminal amino acids of Ptr3, starting from residue 647, with 11 novel residues CWNKNPLSSIN encoded by the Ty1 element.

### *GAP1* Transcription is Induced in Ptr3^647::C…N^

**Figure 3:**
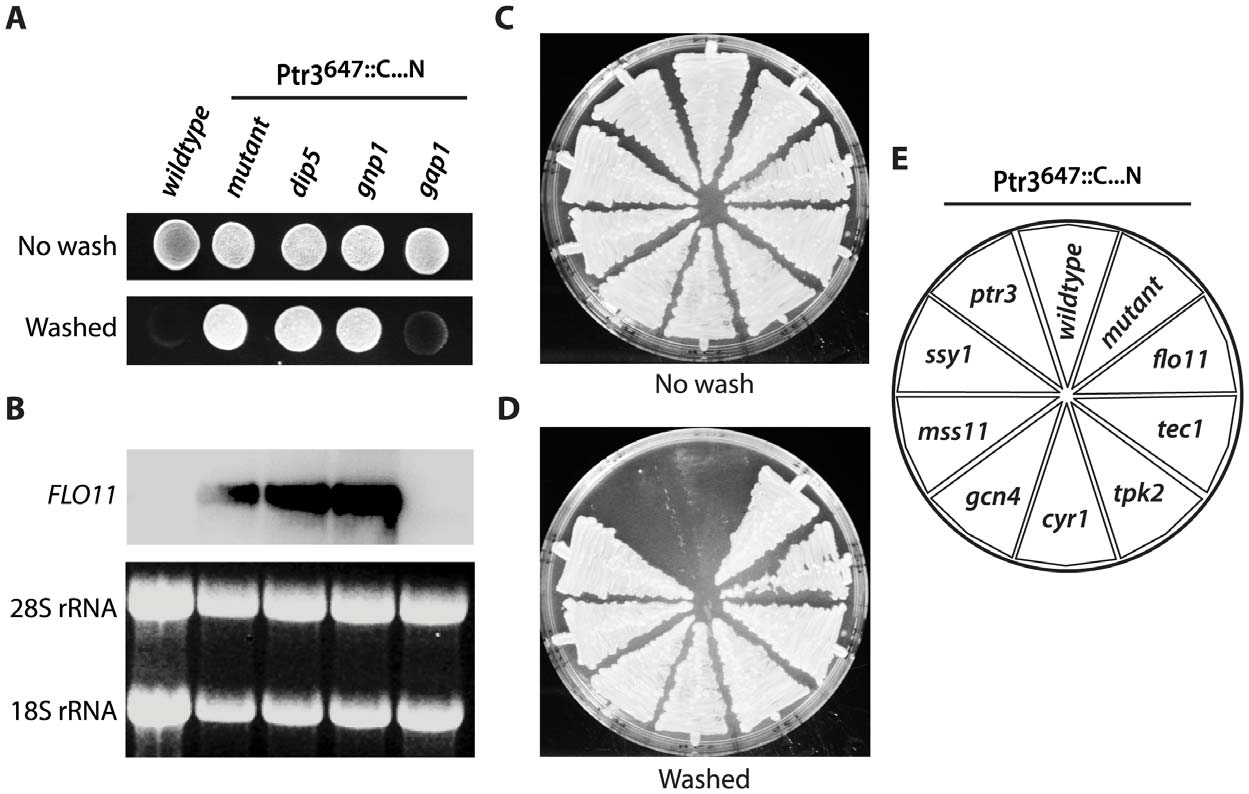
Gap1-dependent *FLO11* expression and invasive growth in Ptr3^647::C…N^. Amino acid permease gene product in invasive growth ability in Ptr3^647::C…N^ was tested in a ura3 PTR3^647::C…N^ background (RB428) by introducing gene deletions dip5 (RB468), gnp1 (RB469) or gap1 (RB547). Cells were grown on rich complex medium (YPD) for 2 day at 30°C (A, upper panel) and flushed with water to expose invasive growth (A, lower panel). Northern blot of FLO11 mRNA for strains in A are shown in B (upper panel). RNA for ribosomal subunits 18S and 28S served as loading controls (B, lower panel). Genes of modulating proteins in invasive growth were deleted in the RB428 mutant: flo11 (RB457), tec1 (RB460), tpk2 (RB461), cyr1 (RB465), gcn4 (RB472), mss11 (RB474), ssy1 (RB477) and ptr3 (RB492). Cells were grown on YPD for 3 days at 30°C (C). The plate was flushed with water to test for invasive growth (D). Distribution of deletion strains in C and D are in E.

Ptr3 is part of the SPS complex that responds to the presence of amino acids in the environment and induces transcription of amino acid transporter genes including *AGP1*, *GNP1* and *DIP5* [13], [17], [18], which encode glutamine transporters. Transcript profiling of the Ptr3^647::C…N^ clone revealed that *DIP5* and other Ptr3-regulated transporter genes (*GNP1*, *PTR2, BAP2, BAP3*, *TAT1*) had strongly reduced mRNA levels relative to the wildtype (Fig. 2D). The general amino acid permease gene *GAP1* on the other hand, had a 10-fold increase in transcription level. The Ptr3^647::C…N^ clone also had increased transcript levels of *FLO* genes, which are involved in cell-surface and cell-cell adhesion and could potentially explain the adhesive phenotype. *FLO5* and *FLO9* were mildly induced while *FLO1* was upregulated 10-fold (Fig. 2D). Northern blot analysis showed that *FLO11* had increased mRNA levels in the Ptr3^647::C…N^ mutant showing that also *FLO11* had been induced (Fig. 3B). We found a clear discrepancy between *FLO11* mRNA levels found in the Northern blot and the array analysis (Fig. 2D). This difference could be caused by binding of unspecific mRNA species to the 60-mer probe used in the array analysis. The 621 bp long probe used for Northern blot analysis was specific for *FLO11*, and hence, we made the assumption that Northern blot analysis was more representative for the *FLO11* mRNA levels.

### *GAP1* is Essential for *FLO11* Expression and Invasive Growth in Ptr3^647::C…N^

To test if increased *GAP1* expression was causative for *FLO* expression and invasive growth we deleted *GAP1*. The *gap1* Ptr3^647::C…N^ mutant lost the ability to grow invasively while deletion of the glutamine transporter genes *DIP5* or *GNP1* in Ptr3^647::C…N^ did not affect invasiveness (Fig. 3A). Northern blot analysis showed that *FLO11* mRNA could not be detected in the *gap1* Ptr3^647::C…N^ mutant (Fig. 3B), suggesting that *GAP1* was essential for *FLO11* expression and invasive growth in this mutant.

### *GAP1* also Induce Invasive Growth Independent of *FLO11*

We next tested if the regulation of invasive growth in the Ptr3^647::C…N^ clone was dependent on *FLO11* and known regulators of *FLO11* [19]. Invasive growth was still maintained after gene-deletion of either *FLO11*, the mitogen-activated protein kinase (MAPK) transcription factor gene *TEC1*, or the PKA-pathway genes *TPK2* and *CYR1*. The same result was obtained when we deleted the transcription factor genes *GCN4* and *MSS11* as well as the SPS-component gene *SSY1* (Fig. 3C–E). Only deletion of the *PTR3^647::C…N^* allele with a *ptr3::KanMX* cassette led to loss of invasive growth. Hence, besides *FLO11*, a second response was responsible for invasive growth in Ptr3^647::C…N^. The response was dependent on high *GAP1* expression and not dependent on the MAPK-, PKA- and SPS-pathways.

### Intracellular Amino Acid Pools are Increased in *PTR3^647::C…N^*

**Figure 4:**
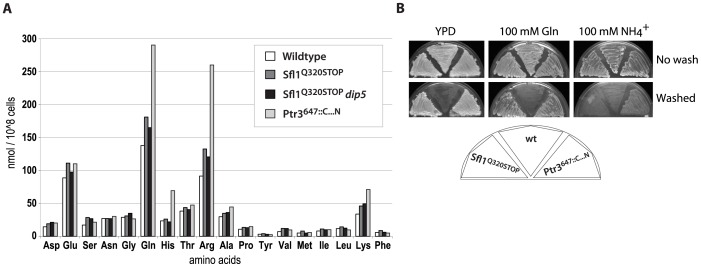
Increased Gln, His, Arg and Lys pools in Ptr3^647::C…N^ and invasive growth of mutants on glutamine minimal medium. (A) shows amino acid concentrations (nmol 10^8^ cells^−1^) in whole cells of ura3 strains isogenic to the wildtype (CEN.PK113-5D), Sfl1^Q320STOP^ (RB20), Sfl1^Q320STOP^ *dip5* (RB317) and Ptr3^647::C…N^ (RB428) after growth to 1–2×10^7^ cells ml^−1^ in liquid YPD. Values shown are representative of three independent experiments. Descendants from glutamine-limited cultures of Sfl1^Q320STOP^ and Ptr3^647::C…N^ were grown as indicated (B) on complex YPD, synthetic minimal medium with 100 mM glutamine or 100 mM ammonium as the sole nitrogen source for 5 days at 30°C. Plates were washed to reveal adhesion.

To explain the role of *GAP1* for invasive growth, we hypothesized that Gap1 could either i) change the balance in the intracellular pools of certain amino acids, ii) affect the activity of invasive growth regulators, and/or iii) act as receptors, eliciting a signal transduction pathway causing invasiveness. To test the first hypothesis, amino acid pool sizes were determined for clones Ptr3^647::C…N^ and Sfl1^Q320STOP^ (Fig. 4A). Compared to the wildtype strain, glutamine pools were almost two-fold higher in the Ptr3^647::C…N^ clone while the pool of the nitrogen-rich vacuolar amino acids lysine, histidine and arginine was as high as 2.5-fold higher than in the wildtype. We hypothesized that high levels of glutamine induced invasive growth. Both Ptr3^647::C…N^ and Sfl1^Q320STOP^ were invasive when grown on medium with 100 mM glutamine as the only nitrogen source, while the wildtype strain remained noninvasive. The strains were not invasive on ammonium (Fig. 4B) suggesting that the effect was glutamine specific.

### *PTR3^647::C…N^* is a Dominant Loss-of-function Allele

The transcript profile of Ptr3^647::C…N^ was similar to transcript profiles of *ptr3* deletion mutants, with severe reductions in transcript levels of SPS-regulated amino acid and peptide transporters *GNP1*, *PTR2* and *DIP5* (Fig 2D; [17]). Hence, *PTR3^647::C…N^* behaves as a loss-of-function allele when expressed on YPD. Reconstitution of the *PTR3^647::C…N^* allele in the wildtype strain (Fig. 2A–C) and mating of the resultant invasive clone with a wildtype *PTR3* strain, CEN.PK110-16D, revealed that the *PTR3^647::C…N^* allele was dominant for invasive growth (data not shown). Moreover, the finding that *SSY1* deletion did not suppress the invasive phenotype of Ptr3^647::C…N^ (Fig. 3C–E) suggested that the *PTR3^647::C…N^* allele is epistatic to *ssy1* and supported that it is dominant. Taken together, these data suggest that the *PTR3^647::C…N^* allele must be a dominant loss-of-function allele.

### *GNP1* and *DIP5* are Essential for *FLO11* Expression and Invasive Growth in Sfl1^Q320STOP^

**Figure 5:**
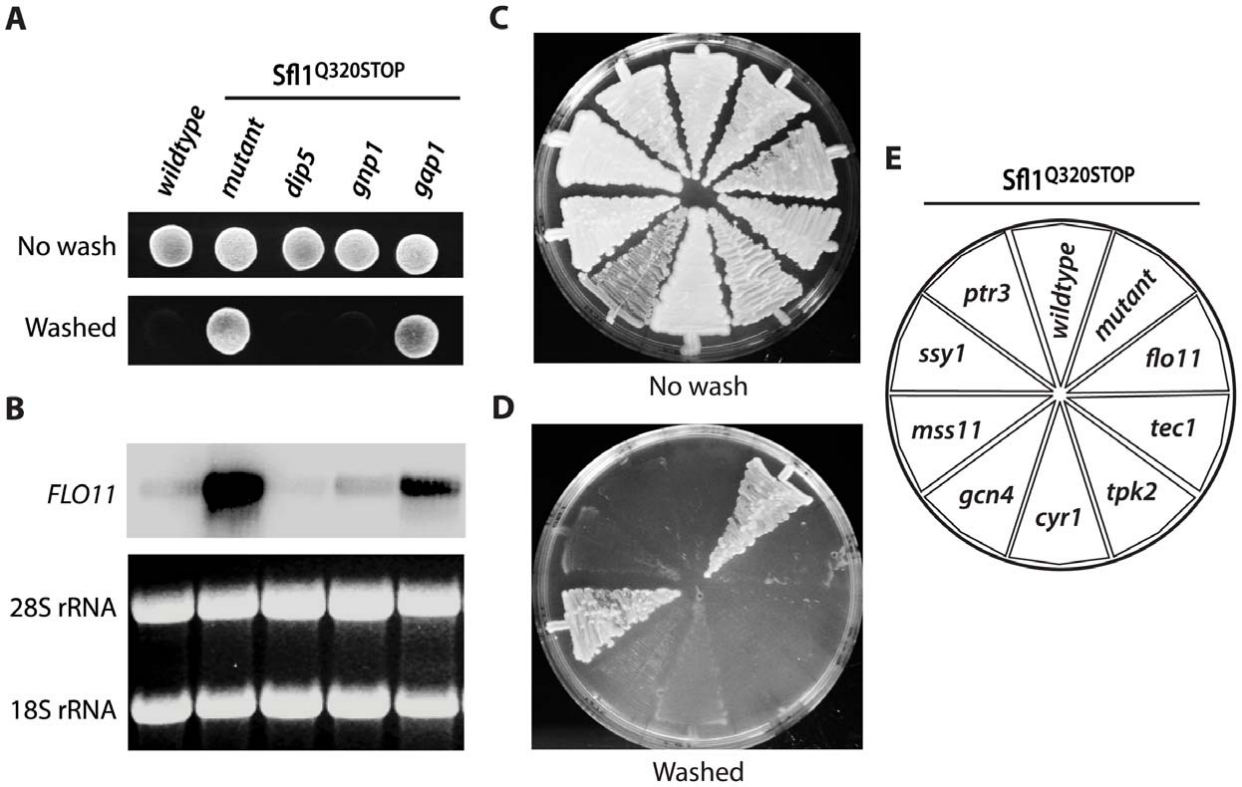
Amino acid permease genes *DIP5* and *GNP1* are essential for invasive growth in Sfl1^Q320STOP^. Amino acid permease genes were deleted in *ura3* Sfl1^Q320STOP^ (RB20) to obtain the mutants dip5 (RB317), gnp1 (RB197) and gap1 (RB531). Strains were grown one day on YPD at 30°C (A, upper panel) and washed to expose invasive growth (A, lower panel). FLO11 mRNA from individual mutants was examined by Northern blotting (B, upper panel) with 18S and 28S rRNA as loading controls (B, lower panel). Regulators of invasive growth were deleted in the RB20 background: flo11 (RB184), tec1 (RB540); tpk2 (RB538), cyr1 (RB201), gcn4 (RB475), mss11 (RB488), ssy1 (RB199) and ptr3 (RB486). After 3 days of growth on YPD (C), plates were flushed with water to reveal invasive growth (D). Mutant Sfl1^Q320STOP^ strains in C and D are shown in E.

The transcript profile of the invasive mutant Sfl1^Q320STOP^ revealed increased transcript levels of the adhesion genes *FLO5*, *FLO9, FLO10* (Fig. 2D) and *FLO11* (Fig. 5B). *SFL1* encodes a PKA-regulated suppressor of *FLO11* [20]–[22] and the *sfl1^Q320STOP^* mutation causes a truncation of the C-terminal part of Sfl1 that normally interacts with the co-suppressor Ssn6 [23]. Because the Ssn6-Sfl1 complex is essential for Sfl1-mediated *FLO11* repression, the *sfl1^Q320STOP^* allele could be responsible for derepression of *FLO11*. The Sfl1^Q320STOP^ clone also carried a SNP in the phosphate transporter gene *PHO90* (Table 1), however the role of the *pho90^T672A^* allele in invasive growth regulation was unclear.

We next tested the role of glutamine transporter genes for invasive growth of Sfl1^Q320STOP^. Both a *gnp1* Sfl1^Q320STOP^ mutant and a *dip5* Sfl1^Q320STOP^ mutant lost their adhesion properties (Fig. 5A) and Northern blot analysis supported these data, showing that *FLO11* mRNA levels were reduced in these strains (Fig. 5B). Thus, *GNP1* and *DIP5* were essential for expression of *FLO11* and invasive growth in a PKA pathway dependent manner. Deletion of *GAP1* in the Sfl1^Q320STOP^ strain had no effect on invasive growth and only led to a partial reduction of *FLO11* expression (Fig. 5B), presumably because (i) *GAP1* was not highly expressed as in Ptr3^647::C…N^ and (ii) *GNP1* and *DIP5* were expressed and responsible for invasive growth.

We also found that *GNP1* and *DIP5* were essential for invasive growth in the *S. cerevisiae* strain ∑1278b. In ∑1278b, deletion of both *GNP1* and *DIP5* was required for loss of invasive growth (Fig. S1). Based on these results, the glutamine transporter genes appear to be a general requirement for invasive growth in the *S. cervisiae* strains ∑1278b and CEN.PK.

### Mutations in *FLO11*, MAPK, SPS and PKA Pathway Genes Suppress Sfl1^Q320STOP^ Invasiveness

Expression of *FLO11* in the Sfl1^Q320STOP^ clone indicated that the *FLO11* promoter was in a PKA pathway regulated derepressed state. Northern blot analysis revealed that *FLO11* was in fact transcribed in the Sfl1^Q320STOP^ clone and deletion of *FLO11* showed that it was responsible for invasive growth (Fig. 5). The absence of Sfl1 repression is known to allow access of transcriptional activators to the *FLO11* promoter [20]–[22]. Individual deletions of the genes *TEC1, TPK2, CYR1, GCN4* and *MSS11*, which encode known regulators of *FLO11* expression [17], [19], led to the loss of invasive growth in all cases, except for the deletion of *MSS11* (Fig. 5C–E). Hence, regulation of invasive growth in the Sfl1^Q320STOP^ clone, CEN.PK background, was similar to regulation in ∑1278b. This result suggests that *GNP1* and *DIP5* induce *FLO11* and invasive growth through identical pathways in the two strain backgrounds involving Gcn4, the MAPK- and PKA-pathways. We tested if regulators of *GNP1* and *DIP5* were also essential for invasive growth. Ssy1-Ptr3-Ssy5 induces the expression of *GNP1* and *DIP5* in response to amino acids [13], [17]. We found that both *PTR3* and *SSY1* were essential for invasive growth in the Sfl1^Q320STOP^ clone (Fig. 5), suggesting that the SPS complex regulates invasive growth through *GNP1* and *DIP5*.

Amino acid pools in the Sfl1^Q320STOP^ mutant and in the Sfl1^Q320STOP^ *dip5* mutant were almost identical to the wildtype strain with a slight increase in the glutamine pool. Thus, Sfl1 and Dip5 did not appear to influence invasive growth through pool sizes (Fig. 4A).

### Dip5 Interacts with Gnp1 and Mep2

**Figure 6:**
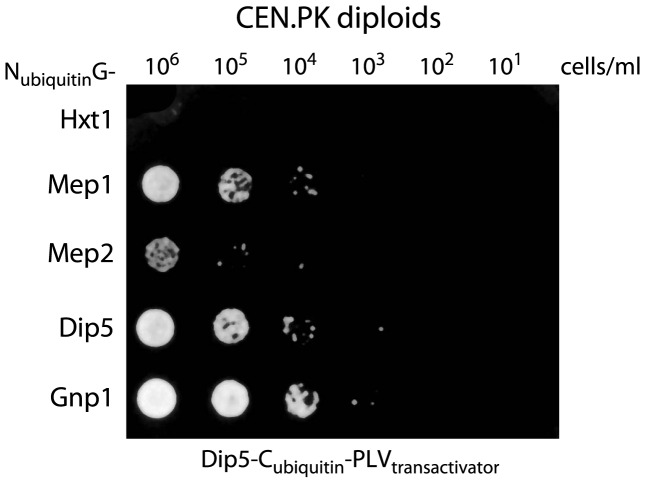
Dip5 interacts with Gnp1 and Mep2. Physical interaction between Dip5 and transporters Hxt1, Mep1, Mep2, Dip5, Gnp1 was tested with the membrane protein-based split-ubiquitin system in the CEN.PK background. Ade^-^ His^-^ cells (THY.AP4) expressing the C-terminal part of ubiquitin fused to Dip5 and the transcriptional activator PLV were mated to Ade^-^ His^-^ cells (THY.AP5) expressing the N-terminal part of ubiquitin fused to either Hxt1, Mep1, Mep2, Dip5 or Gnp1. Interacting protein-pairs formed a functional ubiquitin that released PLV for induction of the HIS3 and ADE2 genes, enabling growth on synthetic complete medium without adenine and histidine.

Interestingly, individual deletions of *DIP5* and *GNP1* resulted in complete loss of *FLO11* expression and invasive growth in Sfl1^Q320STOP^ (Fig. 5A). The finding that two amino acid transporter genes are essential for invasive growth under the same condition indicated corporative behavior between the transporters in the CEN.PK background. We tested the physical interaction between Dip5 and Gnp1, using the split-ubiquitin assay. In accordance with previous findings [24], we found that Dip5 interacts with itself (Fig. 6). Dip5 also interacted with the glutamine transporter Gnp1 and the ammonium permeases Mep1 and Mep2 but did not interact with the glucose transporter Hxt1 (Fig. 6). Physical interaction between Dip5 and Gnp1 might cause a functional dependence for activity of both transporters and explain why deletion of either *DIP5* or *GNP1* led to complete loss of *FLO11* expression in the CEN.PK background.

### *DIP5* is Essential for Biofilm Formation and Pseudohyphal Growth

**Figure 7:**
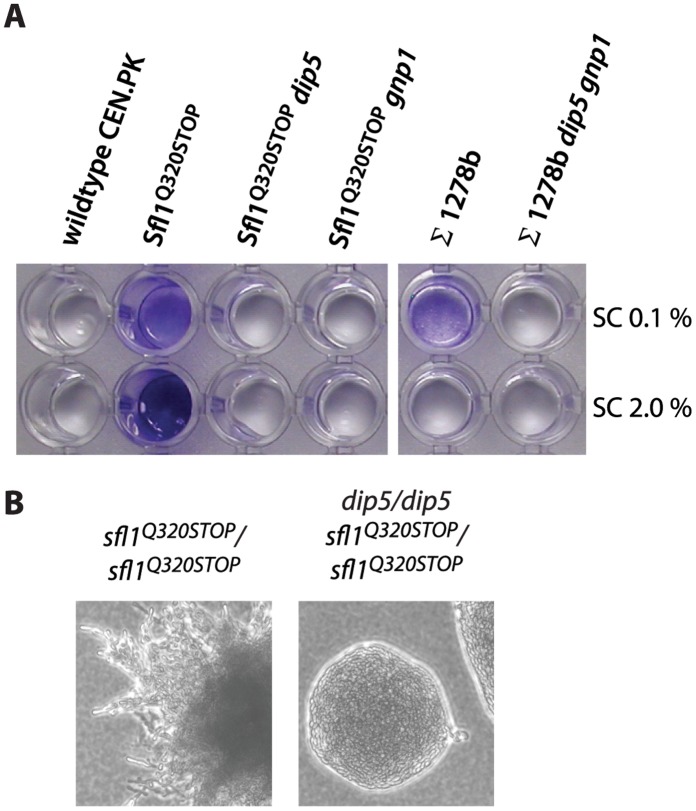
*DIP5* and *GNP1* are essential for biofilm formation and *DIP5* depletion abolishes pseudohyphal differentiation of Sfl1^Q320STOP^. Biofilm formation on a polystyrene surface was tested in the Sfl1^Q320STOP^ trp1 background (RB52) of Sfl1^Q320STOP^ *dip5* (RB317), Sfl1^Q320STOP^ gnp1 (RB196) and compared to ∑1278b strain 10560-2B, ∑1278b *dip5 gnp1* (RB157) and the wildtype strain CEN.PK110-16D. After 24 hours growth, in either synthetic complete 0.1% glucose or synthetic complete 2.0% glucose, biofilms were visualized by crystal violet staining and then washed three times (A). Homozygous diploid strains in the Sfl1^Q320STOP^ background were incubated on low-ammonium SLAD medium for 3 days at 30°C to test for pseudohyphal growth (B).

Invasive growth shares many regulative constituents with biofilm and pseudohyphal growth [19]. We found that the Sfl1^Q320STOP^ mutant formed biofilm on a polystyrene surface and that this was dependent on *DIP5* and *GNP1* (Fig. 7A). A diploid form of Sfl1^Q320STOP^, homozygous for the *sfl1* mutation, formed pseudohyphal growth on synthetic low ammonium medium (SLAD); however, a *sfl1^Q320STOP^*/*sfl1^Q320STOP^ dip5*/*dip5* mutant lost the ability to form pseudohyphae (Fig. 7B). This showed that *DIP5* was essential for the pseudohyphal phenotypes. We attempted to construct *sfl1^Q320STOP^*/*sfl1^Q320STOP^ gnp1*/*gnp1* mutants to test if *GNP1* was required for pseudohyphal growth but were unsuccessful in mating *sfl1^Q320STOP^ gnp1* haploids.

## Discussion

The ammonium transporter Mep2 has been shown to be essential for expression of *FLO11* and filamentous growth of *S. cerevisiae* under nitrogen limitation [8]. Our results suggest that amino acid transporters can have a similar function as Mep2, since they are essential for transcription of the cell surface glycoprotein gene *FLO11*, for biofilm as well as invasive growth in *S. cerevisiae*. We show that *GAP1* is essential for invasive growth and that invasiveness can be maintained even after deletion of *FLO11*. The highly expressed *GAP1* phenotype correlates with an increased amino acid pool and an increased expression of *FLO11* and other *FLO* genes. In cases where *GAP1* is not expressed, we find that *GNP1* and *DIP5* are essential for invasive growth and that they probably exhibit this role through PKA-pathway components and *FLO11* expression.

*GNP1* and *DIP5* induction of *FLO11* is likely to be dependent on an active PKA-pathway, since this regulation is observed in a Sfl1^Q320STOP^ mutant. The transcription factor Sfl1 represses transcription of *FLO11* when activity of the PKA pathway is low [20], [22]. In a ∑1278b background, Sfl1 is removed from the promoter when phosphorylated by an active PKA subunit, Tpk2, leading to *FLO11* transcription [20]–[22]. The PKA pathway is partially inactive in the CEN.PK strain background due to a point mutation in the adenylate cyclase gene, *cyr1^K1876M^* [25]. Therefore, the wildtype CEN.PK strain is likely to have permanent low PKA activity that keeps the *FLO11* promoter in the repressed state. Mutant Sfl1^Q320STOP^ lost the Sfl1 domain that interacts with the co-repressor Ssn6 [23] and the *FLO11* promoter was likely converted to a state amenable to transcription similar to the state found when the PKA pathway is active.

Invasive growth of the Sfl1^Q320STOP^ mutant appeared to be regulated by the same pathways described for the ∑1278b background, including the essential constituents of the PKA-, MAPK- and general amino acid control (GCN) pathways (Fig. 5). Hence, *GNP1* and *DIP5* are likely to be involved in the regulation of one or more of these signaling pathways. The cAMP-PKA and MAPK pathways crosstalk and both are regulated by the G-protein, Ras2 [26] but how this complex signal network is wired in response to *GNP1* and *DIP5* could not be determined by conventional epistasis analysis.

**Figure 8:**
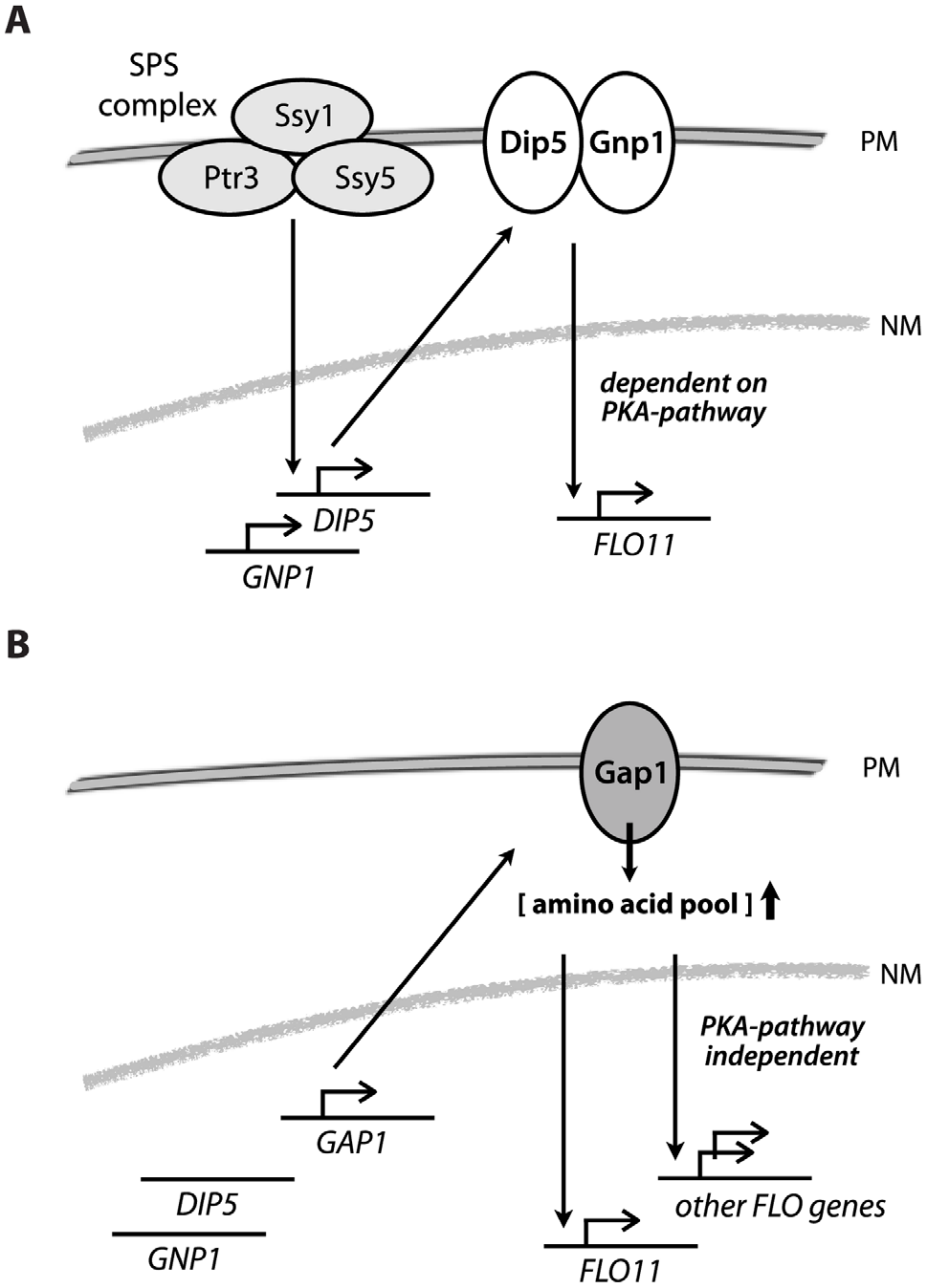
Models of *FLO* gene regulation by amino-acid transporters. Extracellular amino acids induce the SPS complex at the plasma membrane (PM) and the activated complex elicits gene expression of amino acid transporters *DIP5* and *GNP1*. Dip5 and Gnp1 activate *FLO11* transcription in a manner dependent on activity of the PKA-pathway (A). Inactive SPS signaling indirectly leads to increased transcript levels of *GAP1*. Gap1 increases the amino acid pool concentration, which in turn triggers a PKA independent signal for induction of *FLO* genes (B). PM, plasma membrane. NM, nuclear membrane.

The role of the SPS complex (Ptr3, Ssy1 and Ssy5) in regulating invasive growth is so far unclear [17]. Our results suggest that the SPS complex acts on *FLO11* expression by inducing *GNP1*, *DIP5* and potentially other amino acid transporter genes (Fig. 8A model). The model is supported by the fact that the function of *GNP1* and *DIP5* in *FLO11* regulation appears to be conserved between CEN.PK and ∑1278b (Fig. S1).

In the Ptr3^647::C…N^ strain, *GAP1* activity was essential for *FLO11* expression (Fig. 3).

The phenotype of this strain appeared to be similar to previously reported recessive *ptr3* and *ssy1* mutants [17]. Ljungdahl and coworkers found that loss of Ptr3 activity leads to increased *GAP1* transcript levels, reduced levels of *GNP1* and *PTR2* mRNA, invasive growth and increased pools of glutamate, glutamine, histidine, arginine and several other amino acids when grown on YPD [17]. However, in contrast to the recessive *ptr3* alleles, the dominant loss-of-function *PTR3^647::C…N^* allele complemented an *ssy1* deletion, revealing that it was epistatic to *SSY1* (Fig. 3C–E). We propose that Ptr3^647::C…N^ leads to an inactive SPS complex, under the tested conditions, causing reduced transcription of *GNP1* and *DIP5* (Fig. 8B model). *GAP1* transcription is not under direct control of the SPS complex but might be induced in Ptr3^647::C…N^ as a result of altered activity of the TOR-regulated nitrogen catabolite repression pathway [27], giving rise to a higher intracellular amino acid pool. The glutamine transporter genes *GNP1* and *DIP5* are not essential for invasive growth in Ptr3^647::C…N^ probably because these genes have very low expression levels (Fig. 2D).

In addition to Gap1’s role as an amino acid transporter, Gap1 has been reported to induce the PKA pathway, leading to rapid degradation of trehalose and glycogen in nitrogen-starved cells [28]. Hence, Gap1 could be acting on the *FLO11* promoter via the PKA pathway. However, deletion of the PKA pathway genes *CYR1* and *TPK2* in Ptr3^647::C…N^ were not sufficient for loss of invasive growth (Fig. 3C–E). Deletion of other regulatory factors essential for invasive growth in ∑1278b was also not sufficient to eliminate invasive growth by Ptr3^647::C…N^, suggesting a novel *GAP1*-dependent regulatory mechanism. This mechanism acts independent of the PKA-pathway and could regulate transcription of *FLO* genes other than *FLO11* (Fig. 8B model). We found that deletion of *FLO11* was not sufficient to abolish invasive growth, so alternative adhesion proteins must be involved. *S. cerevisiae* contains a number of *FLO* genes that confer cell-cell and cell-surface adhesion [29], [30]. The *FLO1, 5, 9* and *10* genes are normally inactive in laboratory strains though *FLO1*, *FLO5* and *FLO10* are known to have an impact on *S. cerevisiae* adhesion [29], [31]–[33]. A likely candidate is *FLO1*, which was 10-fold induced in the Ptr3^647::C…N^ mutant relative to the wildtype and correlated with a high *GAP1* and *FLO11* expression (Fig. 2D).

In summary, our data demonstrate that amino acid transporters are involved in biofilm development and invasive growth. We propose that at least two modes of action exist on invasive growth regulation; a *FLO11-*dependent modulated by Dip5, Gnp1 and Gap1 and a *FLO11-*independent, regulated by Gap1. The former, Dip5 and Gnp1, are likely amino acid transporters or transceptors that directly activate a cytosolic signaling pathway linked to PKA-pathway components, which in turn induce *FLO11.* Our observations support that both permeases operate downstream of amino acid sensing complex, SPS, for regulation of *FLO11*. The latter, Gap1, may potentially induce invasive growth through increased amino acid pool levels that elicit a novel signal-cascade for gene expression of other *FLO* genes.

## Materials and Methods

### Strains

Strains used are listed in Table 2. The wildtype strain CEN.PK113-7D and the isogenic strains CEN.PK113-5D (*ura3*) and CEN.PK110-16D (*trp1*) were kind gifts from Peter Kötter, Frankfurt am Main, Germany. The two populations of CEN.PK113-7D and two populations of S288c evolved in glucose limitation were kind gifts from Maitreya Dunham, Seattle, USA. The 10560-2B parental strain (∑1276b) and *dip5* (RB598), *gnp1* (RB599) and *gap1* (RB600) mutants in the ∑1276b background were kind gifts from Owen Ryan and Charles Boone, Toronto, Canada [34]. Descendants of CEN.PK113-7D exposed to glutamine limitation for 250 generations were isolated and three clones were chosen for analysis (Table 1). Descendants of CEN.PK113-7D exposed to ammonium limitation for 400 generations were isolated and three clones were chosen for analysis (Table 1).

**Table 2:** *S. cerevisiae* strains and populations used in this study.

Strain	Genotype	Reference/source
Wildtype CEN.PK113-7D	*MAT* **a**	[47]
CEN.PK113-5D	*MAT* **a** *ura3-52* (generated from progenitor CEN.PK113-7D)	[47]
CEN.PK110-16D	*MAT*α *trp1*	[47]
10560-2B (Σ1276*b*)	*MAT* **a** *ura3-52 leu2::hisG his3::hisG*	[48]
THY.AP4	*MAT* **a** *ura3 leu2 lexA::lacZ::trp1 lexA::HIS3 lexA::ADE2*	[46]
THY.AP5	*MAT*α *leu2 trp1 his3 loxP::ade2*	[46]
RB1	*MAT* **a** *sfl1^Q320STOP^ pho90^T672A^*	this study
RB3	*MAT* **a** *PTR3^647::CWNKNPLSSIN^*	this study
RB12	*MAT* **a** Δ*gap1*	this study
RB16	*MAT* **a** *gap1^S388P^ mep2^G352S^*	this study
RB17	*MAT* **a** Δ*gap1 gdh1^D206E^*	this study
RB18	*MAT* **a**	this study
RB20	*MAT* **a** *sfl1^Q320STOP^ pho90^T672A^ ura3*	this study
RB52	*MAT*α *sfl1^Q320STOP^ trp1*	this study
RB157	*∑*1276b *MAT* **a** *ura3-52 leu2::hisG his3::hisG dip5:::KanMX gnp1::loxP*	this study
RB184	*MAT* **a** *sfl1^Q320STOP^ pho90^T672A^ ura3 flo11::KanMX*	this study
RB196	*MAT*α *sfl1^Q320STOP^ trp1 gnp1:: KanMX*	this study
RB197	*MAT* **a** *sfl1^Q320STOP^ pho90^T672A^ ura3 gnp1::KanMX*	this study
RB199	*MAT* **a** *sfl1^Q320STOP^ pho90^T672A^ ura3 ssy1::KanMX*	this study
RB201	*MAT* **a** *sfl1^Q320STOP^ pho90^T672A^ ura3 cyr1::KanMX*	this study
RB209	*MAT*α *sfl1^Q320STOP^ trp1 dip5::KanMX*	this study
RB210	*MAT*α *sfl1^Q320STOP^ ura3 mep2::KanMX*	this study
RB317	*MAT* **a** *sfl1^Q320STOP^ pho90^T672A^ ura3 dip5::KanMX*	this study
RB318	*MAT* **a** *ura3-52 gap1*	this study
RB389	*MAT* **a** *ura3-52 pho90^T672A^*	this study
RB428	*MAT* **a** *PTR3^647::CWNKNPLSSIN^ ura3*	this study
RB457	*MAT* **a** *PTR3^647::CWNKNPLSSIN^ ura3 flo11::KanMX*	this study
RB460	*MAT* **a** *PTR3^647::CWNKNPLSSIN^ ura3 tec1::KanMX*	this study
RB461	*MAT* **a** *PTR3^647::CWNKNPLSSIN^ ura3 tpk2::KanMX*	this study
RB465	*MAT* **a** *PTR3^647::CWNKNPLSSIN^ ura3 cyr1::KanMX*	this study
RB468	*MAT* **a** *PTR3^647::CWNKNPLSSIN^ ura3 dip5::KanMX*	this study
RB469	*MAT* **a** *PTR3^647::CWNKNPLSSIN^ ura3 gnp1::KanMX*	this study
RB472	*MAT* **a** *PTR3^647::CWNKNPLSSIN^ ura3 gcn4::KanMX*	this study
RB474	*MAT* **a** *PTR3^647::CWNKNPLSSIN^ ura3 mss11::KanMX*	this study
RB475	*MAT* **a** *sfl1^Q320STOP^ pho90^T672A^ ura3 gcn4::KanMX*	this study
RB477	*MAT* **a** *PTR3^647::CWNKNPLSSIN^ ura3 ssy1::KanMX*	this study
RB484	*MAT* **a** *ura3-52 gdh1^D206E^*	this study
RB486	*MAT* **a** *sfl1^Q320STOP^ pho90^T672A^ ura3 ptr3::KanMX*	this study
RB488	*MAT* **a** *sfl1^Q320STOP^ pho90^T672A^ ura3 mss11::KanMX*	this study
RB492	*MAT* **a** *PTR3^647::CWNKNPLSSIN^ ura3 ptr3::KanMX*	this study
RB531	*MAT* **a** *sfl1^Q320STOP^ pho90^T672A^ ura3 gap1*	this study
RB538	*MAT* **a** *sfl1^Q320STOP^ pho90^T672A^ ura3 tpk2::KanMX*	this study
RB540	*MAT* **a** *sfl1^Q320STOP^ pho90^T672A^ ura3 tec1::KanMX*	this study
RB547	*MAT* **a** *PTR3^647::CWNKNPLSSIN^ ura3 gap1*	this study
RB567	*MAT* **a** *ura3-52 PTR3^647::CWNKNPLSSIN^*	this study
RB595	*MAT* **a** *ura3-52 sfl1*Δ	this study
RB598	*∑*1276b *MAT* **a** *dip5::KanMX can1::STE2p-SpHIS5 lyp1::STE3p-LEU2 his3::HisG leu2 ura3*	[34]
RB599	*∑*1276b *MAT* **a** *gnp1::KanMX can1::STE2p-SpHIS5 lyp1::STE3p-LEU2 his3::HisG leu2 ura3*	[34]
RB600	*∑*1276b *MAT* **a** *gap1::KanMX can1::STE2p-SpHIS5 lyp1::STE3p-LEU2 his3::HisG leu2 ura3*	[34]

Tiling array analysis of descendants revealed potential SNPs and deletions. SNPs in ORFs were identified by PCR amplification of 500 bp around the potential SNP and Sanger sequencing of the product, while deletions were identified as described previously [35]. Mutations in the three clones from the glutamine limited culture were i) *sfl1^C958T^* and *pho90^A2014G^* (clone Sfl1^Q320STOP^); ii) and *PTR3^1941::Ty1^* (clone Ptr3^647::C…N^, allele *PTR3^647::C…N^*) and iii) YKRCδ11*-Δgap1-*YKRCδ12. The three clones from ammonium limitation carried the mutations: i) *gap1^T1162C^* and *mep2^G1054A^*; ii) YKRCδ11*-Δgap1-*YKRCδ12 and *gdh1^C618G^* and iii) no SNPs were detected in the last clone. The resulting protein modifications are described in Table 1. The *PTR3^647::C…N^* insertion initially appeared as a SNP in *PTR3* in the tiling array analysis but several primer combinations around residue 1941 of *PTR3* failed to give a PCR product. The mutation was identified by digestion of genomic DNA from the Ptr3^647::C…N^ clone with *Xho*I followed by ligation with T4 ligase and inverted primers identical to regions just upstream of residue 1941 for inverse PCR. Sanger sequencing of the PCR product revealed insertion of a long terminal repeat (LTR) identical to *YMRCTy1-4*, *YJRWTy1-2*, *YGRWTy1-1* and *YOLWTy1-1* (http://www.yeastgenome.org/), given that *PTR3^647::C…N^* had an insertion through transposition of one of these Ty1s.

The *PTR3^647::C…N^* allele was reconstituted into the wildtype strain background (CEN.PK113-5D isogenic to CEN.PK113-7D except for *URA3*) by transformation with a chimeric *PTR3^1941::Ty1^* PCR product, using the primers 5′-CATGATACTAGTTGAGGAG-3′ (identical to nucleotides 983–1001 of the *PTR3* ORF) and 5′-tcgaagatgactatgggtttccgacgattcatgaatgagtcGATTCCATTTTGAGGATTCC-3′ (identical to nucleotides 215–234 of the Ty1 LTR and 213143–213173 of chromosome XI downstream of the stop codon of wild type *PTR3*). The PCR product was co-transformed into CEN.PK113-5D with a *URA3* plasmid. A prototrophic transformant able to adhere and invade agar had the correct insertion of the *PTR3^647::C…N^* mutation, identified by PCR and Sanger sequencing. The resultant strain was named RB567. *gdh1^C618G^* and *pho90^A2014G^* were reconstituted into CEN.PK113-5D by co-transformation of a *URA3* plasmid with PCR products carrying *gdh1^C618G^* or *pho90^A2014G^*. Correct clones were identified through prototrophic, invasive growth and Sanger sequencing and the resultant mutants denoted RB484 and RB389. *sfl1^C958T^* was not successfully introduced into CEN.PK113-5D; therefore a full *sfl1* deletion was used as substitute (RB595). YKRCδ11*-gap1-*YKRCδ12 mutants (RB318, RB531, RB547) were selected on D-histidine [36] and YKRCδ11*-gap1-*YKRCδ12 deletions were confirmed by PCR as described [35].

Auxotrophic *ura3* Sfl1^Q320STOP^ and Ptr3^647::C…N^ strains were selected on medium with 5-FOA [37] and the genotype confirmed by complementation with *URA3*-based plasmids to generate strains RB20 and RB428. PCR-based gene deletions were made as described [38], using primers to the *loxP::KanMX::loxP* cassette in pUG6 with 5'-flanking sequences of the 40 bp immediately upstream and downstream of the targeted ORF. Deletion mutants were selected on YPD with 200 mg L^−1^ geneticin. Correct insertion of the pUG6-based PCR fragment was confirmed by diagnostic PCR on genomic DNA from geneticin-resistant clones using primers for the *KanMX* cassette and the promoter sequence of the targeted gene (see Table 2 for deletion strains).

A *MAT*α Sfl1^Q320STOP^ *trp1* strain (RB52) was obtained by mating a *MAT*a *sfl1^Q320STOP^ pho90^T672A^ ura3* strain, RB20, and CEN.PK110-16D and sporulating the resultant diploid. Diploid homozygous mutants were obtained by crossing the haploid mutants RB20 (*MAT* **a** *sfl1^Q320STOP^ pho90^T672A^ ura3*) and RB52 (*MAT*α Sfl1^Q320STOP^ *trp1*). Standard yeast genetics and molecular biology methods were used [37].

### Chemostat Cultivation

CEN.PK113-7D *MAT*a was grown in modified minimal medium at dilution rates of 0.20 h^−1^ as described [39]. Cultures were fed with modified minimal medium containing ammonium (in duplicated culture experiments) or glutamine at a concentration corresponding to 6 mM nitrogen and 250 mM glucose (calculated on nitrogen and carbon atom bases, respectively), which ensured that growth was limited by nitrogen source with all other nutrients in excess. After steady state was reached, cellular dry weight, metabolite concentrations and gas profiles were monitored as described [39] every thirteenth generation. All parameters were constant throughout cultivation including biomass yield, glutamine limitation: 0.22±0.01 g dry weight/g glucose consumed and ammonium limitation: 0.22±0.01 and 0.21±0.01 g dry weight/g glucose consumed. Dry weight was determined in the efflux medium to test if cells accumulated because of flocculation in the bioreactor. Residual concentrations of glutamine were below detection level (<30 µM). In the ammonium-limited chemostat, the residual ammonium concentration was less than 50 µM throughout fermentation. Glucose and nitrogen consumption and metabolites (biomass, ethanol and glycerol) throughout chemostat cultivations showed no changes and the C:N ratio was thus constant over time.

### Culture Media

Media for the chemostat cultivations was as described [39]. Standard yeast media, YPD and SC were as described [37]. All standard media were variations of synthetic dextrose medium with 1.7 g L^−1^ yeast nitrogen base without amino acids and ammonium sulphate, 20 g L^−1^ glucose, and 18 g L^−1^ agar. Low ammonium SLAD contained 0.05 mM ammonium sulphate as sole nitrogen source, while synthetic glutamine dextrose medium was supplemented with 100 mM glutamine as the sole nitrogen source.

### Gene Expression Analysis

Cells grown in liquid YPD were harvested in mid-exponential phase by addition of an equal volume crushed ice and centrifugation. RNA was prepared by acid phenol extraction and hybridized to Agilent 60-mer yeast ORF expression microarrays as described [40]. Data were acquired using an Agilent scanner and extracted with Agilent software using standard settings. Ratios of mRNA levels in the descendants to the progenitor were sorted using a 3-fold cutoff (Fig. S2). The 106 resultant Log2 transformed transcript ratios were clustered by centered Pearson correlation. All raw microarray data are available from the Princeton Microarray Database (http://puma.princeton.edu).

### Tiling Arrays and SNP Identification

Affymetrix Yeast Tiling arrays 1.0 were used. Total genomic DNA was hybridized to probes spaced every 5 bases and the data were analyzed as described [41], [49].

### Northern Blots

Northern blot analysis was performed with RNA from cells in YPD in mid-exponential phase. Total RNA was isolated using an acid-phenol extraction protocol [42] and 10 µg separated by electrophoresis, blotted and hybridized as described [43] with the following modifications: a probe was prepared from a PCR-amplified 621 bp *FLO11* DNA fragment from the 3′-end of *FLO11* (≤60% homologous to other regions in the *S. cerevisiae* genome). The probe was radioactively labeled with [Υ-P^32^]-CTP using a Prime-It II Random Primer Labeling Kit (Agilent Technologies, USA) and purified from unincorporated nucleotides using Illustra ProbeQuan G-50 Micro Columns (GE Healthcare, UK). The membrane was pre-hybridized with Ambion ULTRAhyb (Life Technologies Ltd., UK) for 2 hours at 42°C before addition of labeled probe.

### Intracellular Amino Acid Pools

Intracellular amino acids were extracted with trichloracidic acid as described [44] and quantified by reverse-phase HPLC using an LKB-Alpha Plus amino-acid analyser [45].

### Split-ubiquitin Interaction Assay

The split-ubiquitin interaction assay was essentially as described [46]. *S. cerevisiae* genes were amplified from genomic DNA [S288c] using gene-specific primers acaagtttgtacaaaaaagcaggctctccaaccaccATGX_19–25_-5` ORF and tccgccaccaccaaccactttgtacaagaaagctgggtaX_19–25_-3` strand ORF without stop. The *DIP5* PCR product was cloned *in vivo* by homologous recombination in the THY.AP4 strain, carrying the plasmid pMetYCgate, to form the plasmid pMet-*DIP5*-Cgate. The THY.AP5 strain, carrying the plasmid pNXgate33-3HA, was transformed with PCR products of either *HXT1*, *MEP1*, *MEP2*, *DIP5* or *GNP1* to form strains with the plasmids pN-*HXT1*-gate33-3HA, pN-*MEP1*-gate33-3HA, pN-*MEP2*-gate33-3HA, pN-*DIP5*-gate33-3HA and pN-*GNP1*-gate33-3HA. THY.AP4 and THY.AP5 are CEN.PK derivatives and transformants of THY.AP4 were selected on SC -leucine and transformants of THY.AP5 were selected on SC -tryptophan. Plasmids were isolated and amplified in *Escherichia coli* by standard methods and DNA insertion confirmed by PCR. Protein-protein interactions between Dip5, fused to the C-terminal half of ubiquitin (Dip5-CubPLV), and Hxt1, Mep1, Mep2, Dip5, or Gnp1, fused to the N-terminal half of ubiquitin (NubG-X), were determined by mating strain THY.AP4 (Dip5-CubPLV) to strain THY.AP5 (NubG-X). Diploids were selected on SC-leucine and -tryptophan and interaction between Dip5-CubPLV and each NubG-fusion was determined by growth after 3 days on reporter plates (SC+500 µM methionine and without leucine, tryptophan, adenine and histidine).

### Pseudohyphal Transition, Invasive Growth and Biofilm Formation

Pseudohyphal growth assays were as described [3] and performed using diploid mutants. Invasive and adhesive growth was tested by incubating haploid cells on solid medium at 30°C for 2 days. Plates were photographed and a gentle stream of water used to rinse non-invasive cells from the plate. Plates were dried for 30 minutes and photographed again. Biofilm formation in polystyrene 96-wells plates was tested as described [5]. Biofilm formation assay was conducted with *trp1* auxotrophs (CEN.PK110-16D, RB52, RB157, RB196, RB209, 10560-2B; Table 2).

## Supporting Information

Figure S1 **Amino acid permeases are essential for invasive growth in Sfl1^Q320STOP^, Ptr3^647::C…N^ and** ∑**1276b.** The role of amino acid permeases on invasive growth was tested in several strains: a *ura3* auxotroph version of the CEN.PK wildtype (CEN.PK113-5D), the mutants Sfl1^Q320STOP^ *ura3* (RB20) and Ptr3^647::C…N^ *ura3* (RB428) and the Σ1276b strain (10560-2B). In the *ura3* Sfl1^Q320STOP^ background we deleted *dip5* (RB317), *gnp1* (RB197) and *gap1* (RB531). In the *ura3* Ptr3^647::C…N^ background we deleted *dip5* (RB468), *gnp1* (RB469) and *gap1* (RB547). In the ∑1278b background, we tested invasive growth ability of *dip5* (RB598), *gnp1* (RB599), *gap1* (RB600) and *gnp1 dip5* (RB157). All strains were grown as dot spots on rich complex medium (YPD) for 1 day at 30°C (A) and flushed with water to expose invasive growth (B).
(TIF)

Figure S2 **Microarray analysis of descendants after prolonged nitrogen limitation.** Six single clones were collected after chemostat cultivation with a single limited nitrogen source of either glutamine or ammonium. All other nutrients were in excess. After growth to mid-exponential phase in liquid YPD, microarray analysis was performed. Gene expression data are shown for the glutamine-limited descendants Sfl1^Q320STOP^, Ptr3^647::C…N^ and Δ*gap1* (RB12) and for the ammonium-limited *gap1^S388P^ mep2^G352S^* (RB16), Δ*gap1 gdh^D206E^* (RB17) and no confirmed SNPs for RB18 (-). Asterisk denotes descendants that grow invasively. Transcripts are clustered using Pearson correlation and data are presented as log_2_-transformed ratios. The color code bar illustrates the fold-change in gene expression in descendants relative to the progenitor strain CEN.PK113-7D.
(TIF)
