# Supplementary figures and images for: Amino Acid Transporter Genes Are Essential for FLO11-Dependent and FLO11-Independent Biofilm Formation and Invasive Growth in Saccharomyces cerevisiae
Source: PLoS One. 2012 Jul 26;7(7):e41272. doi: 10.1371/journal.pone.0041272 (PMC3406018; doi:10.1371/journal.pone.0041272)

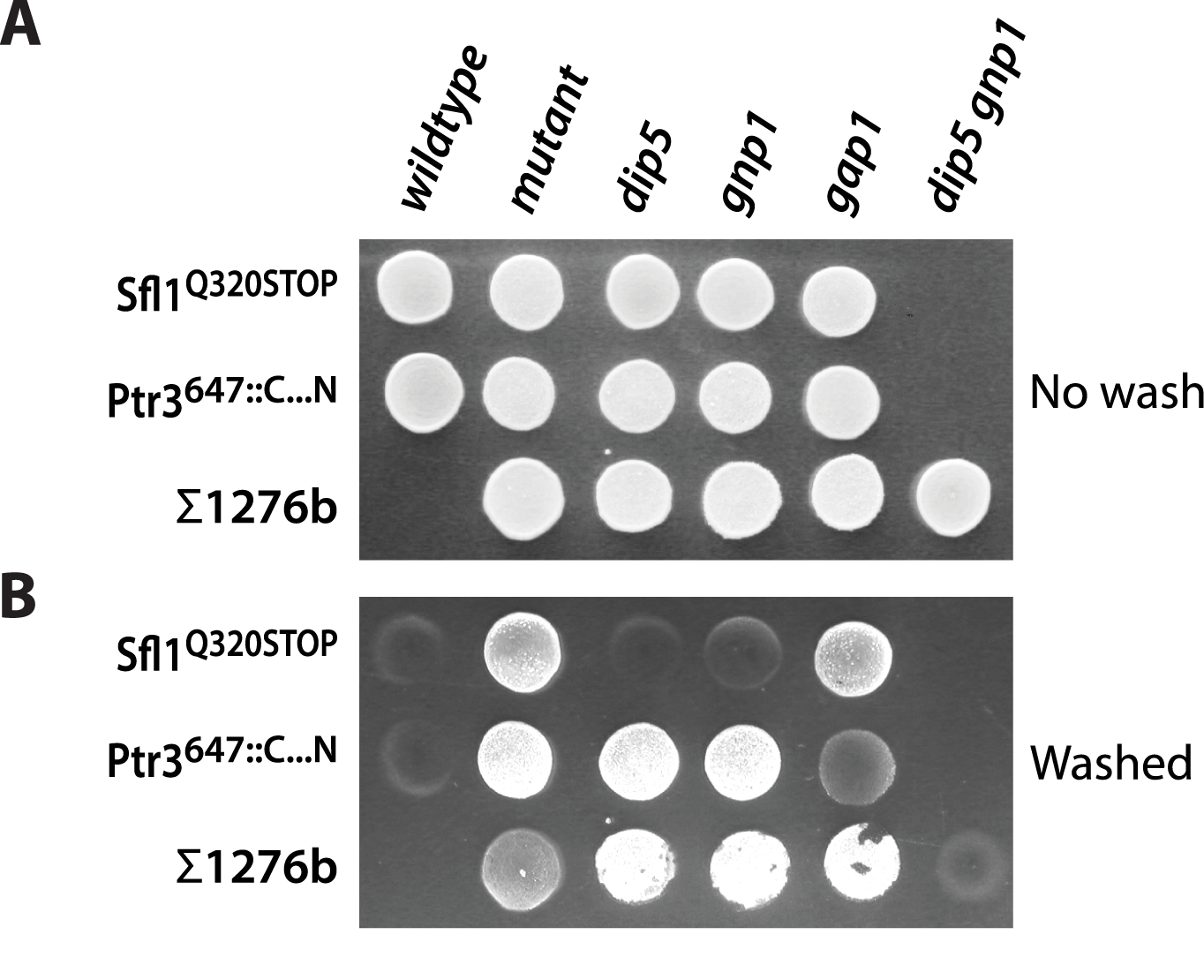

Supplement: Figure S1 — Amino acid permeases are essential for invasive growth in Sfl1Q320STOP, Ptr3647::C…N and ∑1276b. The role of amino acid permeases on invasive growth was tested in several strains: a ura3 auxotroph version of the CEN.PK wildtype (CEN.PK113-5D), the mutants Sfl1Q320STOP ura3 (RB20) and Ptr3647::C…N ura3 (RB428) and the Σ1276b strain (10560-2B). In the ura3 Sfl1Q320STOP background we deleted dip5 (RB317), gnp1 (RB197) and gap1 (RB531). In the ura3 Ptr3647::C…N background we deleted dip5 (RB468), gnp1 (RB469) and gap1 (RB547). In the ∑1278b background, we tested invasive growth ability of dip5 (RB598), gnp1 (RB599), gap1 (RB600) and gnp1 dip5 (RB157). All strains were grown as dot spots on rich complex medium (YPD) for 1 day at 30°C (A) and flushed with water to expose invasive growth (B). (TIF) [file pone.0041272.s001.tif]

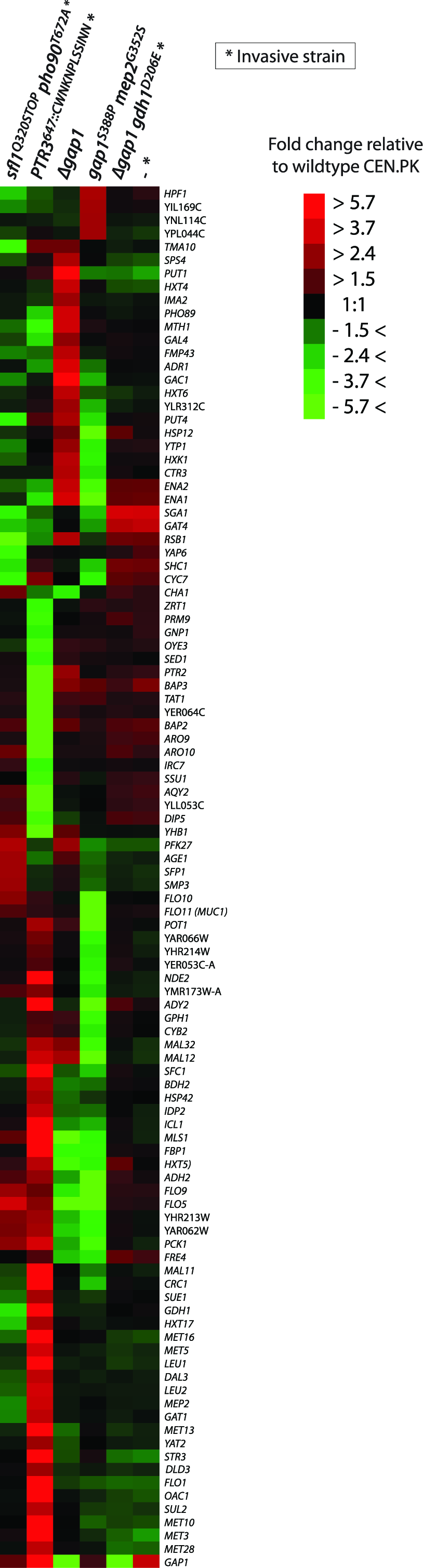

Supplement: Figure S2 — Microarray analysis of descendants after prolonged nitrogen limitation. Six single clones were collected after chemostat cultivation with a single limited nitrogen source of either glutamine or ammonium. All other nutrients were in excess. After growth to mid-exponential phase in liquid YPD, microarray analysis was performed. Gene expression data are shown for the glutamine-limited descendants Sfl1Q320STOP, Ptr3647::C…N and Δgap1 (RB12) and for the ammonium-limited gap1S388P mep2G352S (RB16), Δgap1 gdhD206E (RB17) and no confirmed SNPs for RB18 (-). Asterisk denotes descendants that grow invasively. Transcripts are clustered using Pearson correlation and data are presented as log2-transformed ratios. The color code bar illustrates the fold-change in gene expression in descendants relative to the progenitor strain CEN.PK113-7D. (TIF) [file pone.0041272.s002.tif]
